# Associations between organized sport participation and mental health difficulties: Data from over 11,000 US children and adolescents

**DOI:** 10.1371/journal.pone.0268583

**Published:** 2022-06-01

**Authors:** Matt D. Hoffmann, Joel D. Barnes, Mark S. Tremblay, Michelle D. Guerrero

**Affiliations:** 1 Department of Kinesiology, California State University, Fullerton, California, United States of America; 2 Independent Researcher, Ottawa, Ontario, Canada; 3 Children’s Hospital of Eastern Ontario Research Institute, Ottawa, Ontario, Canada; 4 Department of Pediatrics, University of Ottawa, Ottawa, Ontario, Canada; University of Pennsylvania, UNITED STATES

## Abstract

The purpose of this study was to explore the association between participation in organized sport and a broad array of mental health difficulties among US children and adolescents. The data (cross-sectional) were from Data Release 3.0 (one-year follow-up visits on the full cohort) of the Adolescent Brain Cognitive Development (ABCD) study—a broadly representative sample of 11,235 US children and adolescents aged 9 to 13 years. Parents/guardians provided self-reports of their child’s mental health difficulties using the Child Behavior Checklist. To assess participation in organized sport, children and adolescents were categorized into one of four groups: 1) participation in team sport, 2) participation in individual sport, 3) participation in team and individual sport, and 4) non-sport participation. Participation in team sport compared to non-sport participation was associated with 10% lower anxious/depressed scores, 19% lower withdrawn/depressed scores, 17% lower social problems scores, 17% lower thought problems scores, and 12% lower attention problems scores. Participation in team sport compared to non-sport participation was also associated with 20% lower rule-breaking behavior scores for females (compared to males). Conversely, participation in individual sport compared to non-sport participation was associated with 16% higher anxious/depressed scores, 14% higher withdrawn/depressed scores, 12% higher social problems scores, and 14% higher attention problems scores. Participation in both team and individual sport compared to non-sport participation was associated with 17% lower rule-breaking behavior scores for females (compared to males). Results indicate that team sport participation was associated with fewer mental health difficulties, whereas individual sport participation was associated with greater mental health difficulties. The findings complement previous research suggesting that team sport participation may be a vehicle to support child and adolescent mental health. Additional research is needed to determine to what extent, and under what circumstances, participation in individual sport may be problematic for younger cohorts.

## Introduction

The World Health Organization (WHO) has cited poor mental health as one of the leading predictors of disability and economic burden [1]. Among children and adolescents, mental health difficulties are prevalent across the world [2]. One factor that may protect against the development of mental health difficulties is participation in organized youth sport [3,4]. Paradoxically, some research has linked youth sport participation with adverse outcomes such as anxiety and burnout [5]. In this study, we adopt the view that ‘organized youth sport’ encompasses both child and adolescent participation in organized physical activity, is generally directed by adult or youth leaders, involves rules and formal practice, and is competitive in nature [6]. Because rates of organized youth sport participation are relatively high in some countries but inadequate in others [7], continued efforts to promote sport participation and determine which types of organized sport best support child and adolescent mental health are warranted. The purpose of this study was to extend the literature by exploring the link between participation in organized sport and a comprehensive catalogue of mental health difficulties among US children and adolescents.

Mental health is broadly defined as a state of well-being where a person realizes their own abilities, can cope with the common stresses of life, can work in a productive manner and is able to contribute to their community [8]. Extending this definition, mental health can be understood as a state that involves both the absence of mental illness/difficulties but also the presence of mental health [9]. There is growing interest in studying the link between sport participation and mental health, though researchers have primarily focused on mental health outcomes with elite athletes [10]. Nonetheless, participation in organized youth sport is beneficial across various mental health indices. Results of a recent meta-analysis indicated that youth who played organized sport had lower symptoms of anxiety and depression compared to those who did not participate in sport [4]. Further, children who maintained sport participation between the ages of 8 and 10 years had greater health-related quality of life scores [11] and lower rates of psychological difficulties [12] at age 10 compared with children who discontinued sport by age 10.

Participation in sport may promote child and adolescent mental health because of the many fruitful opportunities to build social relationships and friendships, which can help foster a sense of belongingness within the athletic context [13]. Social interactions and feelings of relatedness with others are inherent aspects of team sport participation. Consequently, it is not surprising that involvement in organized youth team sport has been associated with better mental health [14]. Compared to non-sport participants, individuals who maintained team sport participation from adolescence (11–16 years) through to young adulthood were less stressed and had lower odds of experiencing panic disorder symptoms [15]. Participation in team sport during the adolescent years has also been associated with fewer symptoms of depression and anxiety at later time points—but this pattern did not hold for those engaged in individual sport [13,14]. A systematic review concluded that child and adolescent participation in team sport appeared to be associated with improved psychosocial health compared to individual activities [3]. Taken together, studies seem to illuminate the advantages of team sport participation, yet evidence suggests children and young people involved in individual sport still generally benefit from decreased mental health difficulties in comparison to those who do not participate in organized sport [16,17].

While research findings have consistently indicated the potential protective role of organized youth sport participation against the emergence of mental health difficulties, further investigation of the relationship between sport participation and child and adolescent mental health is needed for several reasons. First, despite evidence favoring organized youth sport participation, involvement in youth sport has been associated with negative outcomes such as stress, anxiety, maltreatment and abuse, and burnout [5]. Sport participation in the adolescent years has also been linked with increased odds of alcohol use disorder and bulimia [18]. There are both environmental and personal factors which serve as antecedents to such negative outcomes. For instance, high training volumes and frequent intense competition (environmental) as well as perfectionism and low self-esteem (personal) have been associated with burnout among youth athletes [19]. Coaching climates that emphasize winning or encourage immoral actions may also promote aggressive behaviors among athletes [20]. Thus, continued research is needed to tease apart which categories of sport participation (and by extension their environments) relate to mental well/ill-being. Second, much of the sport literature has focused on conventional mental health indices (e.g., anxiety and depression), and researchers have urged for broader investigations of youth mental health that include the assessment of emotional and behavioral difficulties [13]. Third, Vella [5] called for more research examining moderating factors that may explain which groups of individuals are more or less likely to experience mental health benefits from youth sport participation. There may be some differences between males and females as it relates to youth sport participation and mental health;(e.g., [4,13]) thus, further consideration of sex effects may clarify this relationship. Fourth, the incidence of mental health disorders among children and adolescents has been increasing over the decades [21]. This highlights the importance of analyzing recently collected data that are reflective of contemporary trends in society. Although findings related to organized youth sport participation and indices of mental health continue to emerge, some are based on data collected many years ago [22]. In this study we draw on a recently collected, large-scale dataset.

The purpose of this study was to explore the association between participation in organized sport and a broad array of mental health difficulties among US children and adolescents. Based on previous research highlighting the benefits of *team* sport participation for mental health,(e.g., [3,15,23]) we hypothesized that children and adolescents engaged in organized team sport would report a lower incidence of mental health difficulties compared to those not engaged in organized sport. Similarly, we expected that children and adolescents engaged in organized *individual* sport would report a lower incidence of mental health difficulties compared to those not engaged in organized sport—though we predicted smaller effect sizes relative to team sport participation. This hypothesis was based on research showing that team sport participation seems to have a stronger association with favorable mental health outcomes than individual sport participation. (e.g., [16,17]). Finally, we hypothesized that children and adolescents engaged in both *team and individual* sport would report a lower incidence of mental health difficulties compared to non-sport participants, but we made no predictions concerning effect sizes relative to team-only or individual-only sport participation. All analyses explored the potential moderating effect of participant sex.

## Material and methods

### Study design and participants

The data (cross-sectional) were from Data Release 3.0 (wave 2 released in 2020; one-year follow-up visits on the full cohort) of the Adolescent Brain Cognitive Development (ABCD) study—a broadly representative sample of 11,235 US children and adolescents aged 9 to 13 years [24]. Based on the World Health Organization’s definition [25], in this study we define children as those aged 9 years and adolescents as those aged 10–13 years. The ABCD study is an ongoing, longitudinal study examining brain development and health in children aged 9–10 years through early adulthood. The study has a particular focus on brain maturation as it relates to social, emotional, and cognitive development. Data for this study are being collected across 21 sites throughout the United States, on a biennial-to-annual basis, over a 10-year period. Details on the sample, recruitment procedures, measures, and compensation are reported elsewhere [26,27]. Ethics clearance was obtained from all relevant institutional research ethics boards. Informed written consent was obtained from parents/guardians; written assent was obtained from participating children and adolescents.

### Measures

#### Exposures

Parents/guardians were provided with a lengthy list of activities (sports, music, hobbies) and were asked to indicate their child’s lifetime involvement in each. Response options were “yes” or “no” for each activity. For each activity selected, parents/guardians were asked: *“Was any of this activity part of an organized team*, *group*, *band*, *orchestra*, *chorus*, *or program at school*?*”* and “*Was any of this activity part of an organized team*, *group*, *band*, *orchestra*, *chorus*, *or program outside of school*?*”* Responses options were “yes” or “no” for each context. Children and adolescents who participated in organized sport at school, outside of school, both at school and outside of school, as well as those who did not participate in organized sport, were retained in this study. As school sport can be quite competitive in the US, we felt it was appropriate to include sports played within this context. We deemed some activities to be more reflective of strictly physical activity than competitive sport (e.g., yoga) and thus they were not included in this study. Participation in organized activities that were not considered physical in nature (e.g., chess) were not included in this study.

To address this study’s main research question, children and adolescents were categorized into one of four groups based on their involvement in organized sport: 1) participation in team sport only (e.g., volleyball, soccer, basketball), 2) participation in individual sport only (e.g., gymnastics, tennis, wrestling), 3) participation in team and individual sport, and 4) non-sport participation. Team sports are those where athletes work together towards a shared group goal, whereas individual sports are those where athletes compete individually against other athletes for the achievement of primarily personal goals [17]. One organized sport activity (i.e., swimming/water polo) contained two sports that differed significantly in their level of task interdependence, making categorization more difficult. However, because water polo fails to make the list of core youth sports in the US [28], we assumed that a majority of children and adolescents involved in organized swimming/water polo participated in swimming (individual task). A sensitivity analysis with swimming/water polo removed resulted in an overall pattern of results that was consistent with those presented in this study.

#### Outcomes

The Child Behavior Checklist (CBCL) is a parent-rated measure that assesses a broad range of emotional and behavioral syndromes, or mental health difficulties, among children and adolescents aged 6 to 18 years [26,29]. The CBCL comprises over 100 items, which assess eight syndrome scales: anxious/depressed (e.g., “fears doing bad”), withdrawn/depressed (e.g., “rather be alone”), somatic complaints (e.g., “nightmares”), social problems (e.g., “unliked”), thought problems (e.g., “hears things”), attention problems (e.g., “acts too young”), rule-breaking behavior (e.g., “lacks guilt”), and aggressive behavior (e.g., “attacks people”). Parents/guardians answered items on a 3-point scale: not true (0), somewhat/sometimes true (1), or very true/often true (2). Following recommendations by Achenbach and Rescorla [29], we used raw rather than standardized scores in all analyses. Higher scores represent greater mental health difficulties. The CBCL’s scores have demonstrated evidence of reliability (α = .78 to .94) [29], and factorial validity evidence has been reported in 30 different societies (e.g., Root Mean Square Error of Approximation ranged from .026 to .055 across all societies) [30].

#### Covariates

We adjusted for several potential confounding variables, including age, sex, race/ethnicity, and family household income (1 = <US$5,000; 2 = $5,000−11,199; 3 = $12,000−15,999; 4 = $16,000−24,999; 5 = $25,000−34,999; 6 = $35,000−49,999; 7 = $50,000−74,999; 8 = $75,000−99,999; 9 = $100,000−199,999; and 10 = ≥$200 000). To determine recent sport participation, and because frequency/volume of sport participation is associated with fewer depressive symptoms [4] and mental health difficulties [31] among adolescents, we assessed the number of hours that children and adolescents participated in organized sport. Sport volume (in hours) was calculated by multiplying the number of months a child or adolescent participated in an organized sport since the previous data collection visit (roughly 12 months prior to the one-year follow-up visit), by the number of days per week they participated in that sport, by the number of minutes they participated in that sport per session, divided by 60. The number of hours of sport participation across all organized sports were combined to produce a total sport volume value. For any given sport, children and adolescents who indicated previous participation but who reported zero hours of engagement (sport volume) within roughly the past 12 months were assigned a value of zero (hours) and coded as non-sport participants. Finally, consistent with previous studies,(e.g., [16,17]) we controlled for overall physical activity levels. Self-report physical activity data were not available in the one-year follow-up visit (i.e., wave 2); thus, we used physical activity data from the baseline visit (*M*_age_ = 9.92; *SD* = 0.63). Physical activity was measured through the following question: “*During the past 7 days*, *on how many days were you physically active for a total of at least 60 minutes per day*?*”*

### Statistical analyses

Statistical analyses were performed using RStudio Version 1.4.1103. Missing data were <1% for all variables in the analyses except for family income where missingness was 8%. List-wise deletion was applied by the models. Given the distribution of the CBCL scores (positively skewed, over-dispersed), we treated the data as count variables and fit negative binomial regression models accordingly. Negative binomial regression was conducted using the glmmTMB R package [32], and we used a mixed-effects modeling approach to account for the non-independence of observations/clustering of data (i.e., data collection sites). Incidence rate ratios (IRRs) were calculated as estimates of effect size, and we examined 95% confidence intervals (CIs) to determine statistical significance. Conditional R^2^ values were used to estimate variance (i.e., fixed and random effects) explained by each model. Non-sport participation served as the reference category for the main analyses (i.e., comparison of sport participation types to non-sport participation). All statistical models included the covariates. Interaction effects between sport type and participant sex were also tested in all models.

## Results

Means, standard deviations, and frequencies are displayed in Table 1. There was adequate representation of sport types across the sample, with non-sport participation representing the greatest proportion of children and adolescents (33.6%) and both team and individual sport participation representing the smallest proportion of children and adolescents (15.6%). Males (52.3%) and females (47.7%) were (relatively) equally represented. Most participants identified as White (53.3%), followed by Hispanic (19.8%), and Black (14.2%). Aggressive behavior, attention problems, and anxious/depressed were the most frequently reported syndromes.

**Table 1:** Descriptive statistics of study participants.

Variable	N	Proportion (%)	Missing	Mean	SD
*Demographic variables*	–	–	–	–	–
Age (years; range 9–13)	11235	–	0	10.9	0.64
Sex	11235	–	0	–	–
Boy	5879	52.3	–	–	–
Girl	5356	47.7	–	–	–
Race/ethnicity	11228	–	7	–	–
White	5986	53.3	–	–	–
Hispanic	2226	19.8	–	–	–
Black	1597	14.2	–	–	–
Asian	243	2.2	–	–	–
Other	1176	10.5	–	–	–
Family income (range 1–10)	10324	–	911	7.3	2.38
Physical activity (days; range 0–7)	11216	–	19	3.5	2.31
Sport volume (hours; range 0–351)	11189	–	46	24.0	32.53
*Predictors*	–	–	–	–	–
Sport type	11235	–	0	–	–
No sports	3771	33.6	–	–	–
Team sports	3348	29.8	–	–	–
Individual sports	2366	21.1	–	–	–
Team + individual sports	1750	15.6	–	–	–
*Outcome variables*	–	–	–	–	–
Anxious/depressed (range 0–22)	11191	–	44	2.5	3.07
Withdrawal/depressed (range 0–14)	11191	–	44	1.1	1.78
Somatic complaints (range 0–18)	11191	–	44	1.5	1.95
Social problems (range 0–19)	11191	–	44	1.5	2.18
Thought problems (range 0–20)	11191	–	44	1.6	2.22
Attention problems (range 0–19)	11191	–	44	2.9	3.43
Rule-breaking behavior (range 0–20)	11191	–	44	1.1	1.82
Aggressive behavior (range 0–33)	11191	–	44	3.1	4.19

Proportions of children and adolescents who participated in organized sports across the three contexts (i.e., school only, outside of school only, or both in and outside school) are reported in Table 2. Generally, regardless of sport, most participants competed outside of school, followed by participation in and outside of school, and within school only. This study’s results are therefore perhaps most relevant to children and adolescents who compete in sport outside of the school setting. Participation in track-and-field/running/cross-country was a notable exception, whereby the majority of children and adolescents who participated in this sport category competed only within a school setting.

**Table 2:** Number of participants stratified by sport and context.

Sport	School only	Outside school only	In and outside school
*Team*	**-**	**-**	**-**
Soccer	109 (6.9%)	1054 (66.8%)	416 (26.3%)
Baseball	34 (3.5%)	710 (72.2%)	240 (24.4%)
Basketball	224 (16.7%)	678 (50.7%)	436 (32.6%)
Field hockey	10 (25.6%)	21 (53.8%)	8 (20.5%)
Football	67 (11.0%)	370 (60.6%)	174 (28.5%)
Ice hockey	2 (1.5%)	114 (84.4%)	19 (14.1%)
Lacrosse	-	159 (100.0%)	‐
Rugby	‐	15 (88.2%)	2 (11.8%)
Volleyball	48 (22.9%)	108 (51.4%)	54 (25.7%)
*Individual*	-	-	-
Ballet/dance	101 (13.0%)	465 (60.1%)	208 (26.9%)
Gymnastics	22 (4.2%)	424 (80.9%)	78 (14.9%)
Horseback riding	1 (1.4%)	57 (82.6%)	11 (15.9%)
Martial arts	10 (2.1%)	390 (82.5%)	73 (15.4%)
Wrestling	8 (11.0%)	50 (68.5%)	15 (20.5%)
Swimming/water polo	49 (7.7%)	478 (74.9%)	111 (17.4%)
Tennis	16 (11.2%)	92 (64.3%)	35 (24.5%)
Track/running/cross-country	154 (52.9%)	55 (18.9%)	82 (28.2%)
*Team + individual*	-	-	-
Soccer	71 (8.9%)	574 (71.8%)	155 (19.4%)
Baseball	23 (4.5%)	371 (72.2%)	120 (23.3%)
Basketball	140 (20.5%)	345 (50.6%)	197 (28.9%)
Field hockey	6 (26.1%)	16 (69.6%)	1 (4.3%)
Football	28 (13.7%)	116 (56.9%)	60 (29.4%)
Ice hockey	‐	41 (83.7%)	8 (16.3%)
Lacrosse	‐	107 (100.0%)	‐
Rugby	‐	9 (100.0%)	‐
Volleyball	47 (28.7%)	84 (51.2%)	33 (20.1%)
Ballet/dance	42 (12.2%)	222 (64.7%)	79 (23.0%)
Gymnastics	3 (1.2%)	205 (83.7%)	37 (15.1%)
Horseback riding	1 (1.6%)	57 (93.4%)	3 (4.9%)
Martial arts	4 (1.6%)	218 (84.5%)	36 (14.0%)
Wrestling	11 (11.7%)	57 (60.6%)	26 (27.7%)
Swimming/water polo	25 (4.0%)	496 (80.0%)	99 (16.0%)
Tennis	13 (7.8%)	130 (78.3%)	23 (13.9%)
Track/running/cross-country	219 (54.9%)	109 (27.3%)	71 (17.8%)

Spearman correlations among the major study variables can be viewed in the S1 Fig. The results of the main analyses are reported in Tables 3–5. Overall, there were fewer mental health difficulties among females compared to males, and among those who identified as Black and Asian compared to those who identified as White. Greater household income was a stable predictor of fewer mental health difficulties. In terms of key findings, participation in team sports compared to non-sport participation was associated with 10% lower anxious/depressed scores (IRR = .90; 95% CI [.83, .97]), 19% lower withdrawn/depressed scores (IRR = .81; 95% CI [.74, .89]), 17% lower social problems scores (IRR = .83; 95% CI [.77, .91]), 17% lower thought problems scores (IRR = .83; 95% CI [.77, .90]), and 12% lower attention problems scores (IRR = .88; 95% CI [.82, .95]). Participation in team sports compared to non-sport participation was also associated with 20% lower rule-breaking behavior scores (IRR = .80; 95% CI [.68, .93]) for females (compared to males). Conversely, participation in individual sports compared to non-sport participation was associated with 16% higher anxious/depressed scores (IRR = 1.16; 95% CI [1.05, 1.27]), 14% higher withdrawn/depressed scores (IRR = 1.13; 95% CI [1.01, 1.28]), 12% higher social problems scores (IRR = 1.12; 95% CI [1.01, 1.25]), and 14% higher attention problems scores (IRR = 1.14; 95% CI [1.04, 1.25]). Participation in both team and individual sports compared to non-sport participation was associated with 17% lower rule-breaking behavior scores (IRR = .83; 95% CI [.70, .99]) for females (compared to males). Models accounted for approximately 3–9% of the variance in mental health difficulty scores.

**Table 3:** Associations between types of sport participation and mental health difficulties.

	Anxious/depressed		Withdraw/depressed		Somatic complaints	
*Predictors*	*Incidence Rate Ratios*	*95% CI*	*Incidence Rate Ratios*	*95% CI*	*Incidence Rate Ratios*	*95% CI*
Intercept	**2.480**	1.710–3.596	0.653	0.403–1.057	1.508	0.989–2.301
Age (years)	1.018	0.986–1.052	**1.116**	1.070–1.164	1.021	0.984–1.060
Girl (ref: Boy)	1.036	0.964–1.112	0.933	0.856–1.017	**1.104**	1.019–1.196
Black (ref: White)	**0.652**	0.601–0.707	**0.788**	0.715–0.869	**0.765**	0.701–0.835
Hispanic (ref: White)	0.949	0.889–1.012	0.946	0.871–1.028	0.947	0.880–1.019
Asian (ref: White)	**0.726**	0.615–0.857	**0.766**	0.616–0.952	**0.641**	0.524–0.784
Other (ref: White)	0.960	0.895–1.029	1.055	0.965–1.154	1.061	0.982–1.147
Household income	0.993	0.982–1.004	**0.955**	0.943–0.968	**0.971**	0.960–0.983
Physical activity	**0.991**	0.981–1.000	**0.966**	0.955–0.978	0.998	0.987–1.008
Sport volume (hours)	**0.998**	0.998–0.999	**0.995**	0.994–0.997	**0.998**	0.997–0.999
Team sports	**0.895**	0.830–0.965	**0.811**	0.737–0.894	0.953	0.875–1.038
Individual sports	**1.158**	1.054–1.271	**1.139**	1.013–1.280	1.013	0.907–1.132
Team + individual sports	1.033	0.937–1.139	0.893	0.783–1.018	0.982	0.876–1.101
Sex * team sports	1.029	0.921–1.149	0.983	0.850–1.138	0.961	0.848–1.088
Sex * individual sports	0.893	0.795–1.003	0.869	0.750–1.006	0.993	0.867–1.137
Sex * team + individual sports	0.992	0.876–1.123	1.006	0.850–1.191	1.045	0.905–1.205
**Variance**						
Conditional R^2^	0.039		0.066		0.027	

*Note*. Statistically significant values (*p* < .05) are in bold.

**Table 4:** Associations between types of sport participation and mental health difficulties.

	Social problems		Thought problems		Attention problems	
*Predictors*	*Incidence Rate Ratios*	*95% CI*	*Incidence Rate Ratios*	*95% CI*	*Incidence Rate Ratios*	*95% CI*
Intercept	**6.628**	4.286–10.249	**3.795**	2.518–5.719	**7.033**	4.801–10.303
Age (years)	**0.929**	0.893–0.965	0.969	0.934–1.004	0.969	0.937–1.002
Girl (ref: Boy)	**0.907**	0.838–0.982	**0.766**	0.708–0.829	**0.672**	0.625–0.723
Black (ref: White)	**0.909**	0.834–0.991	**0.703**	0.645–0.767	**0.919**	0.851–0.992
Hispanic (ref: White)	0.956	0.887–1.031	**0.785**	0.728–0.846	0.943	0.880–1.009
Asian (ref: White)	**0.689**	0.559–0.850	**0.629**	0.517–0.765	**0.717**	0.601–0.857
Other (ref: White)	**1.093**	1.007–1.185	1.000	0.927–1.079	**1.116**	1.040–1.197
Household income	**0.937**	0.926–0.948	**0.973**	0.962–0.985	**0.971**	0.961–0.982
Physical activity	**0.983**	0.972–0.994	0.991	0.981–1.001	**0.976**	0.967–0.986
Sport volume (hours)	**0.997**	0.996–0.998	**0.998**	0.997–0.999	**0.997**	0.996–0.998
Team sports	**0.834**	0.765–0.910	**0.833**	0.768–0.902	**0.883**	0.821–0.950
Individual sports	**1.122**	1.008–1.250	1.102	0.998–1.217	**1.137**	1.039–1.245
Team + individual sports	0.937	0.833–1.054	0.978	0.881–1.086	0.976	0.885–1.076
Sex * team sports	1.026	0.900–1.171	1.131	0.999–1.280	1.003	0.891–1.129
Sex * individual sports	0.888	0.776–1.016	0.975	0.858–1.108	0.937	0.832–1.056
Sex * team + individual sports	1.011	0.869–1.177	1.021	0.888–1.174	1.056	0.926–1.206
**Variance**						
Conditional R^2^	0.058		0.050		0.071	

*Note*. Statistically significant values (*p* < .05) are in bold.

**Table 5:** Associations between types of sport participation and mental health difficulties.

	Rule-breaking behavior		Aggressive behavior	
*Predictors*	*Incidence Rate Ratios*	*95% CI*	*Incidence Rate Ratios*	*95% CI*
Intercept	**2.594**	1.599–4.210	**5.875**	3.970–8.693
Age (years)	0.99	0.95–1.03	0.99	0.96–1.03
Girl (ref: Boy)	**0.729**	0.665–0.798	**0.867**	0.804–0.935
Black (ref: White)	1.073	0.979–1.177	**0.844**	0.780–0.913
Hispanic (ref: White)	0.957	0.878–1.044	0.935	0.872–1.002
Asian (ref: White)	**0.608**	0.472–0.784	**0.629**	0.520–0.761
Other (ref: White)	**1.169**	1.070–1.278	1.033	0.960–1.112
Household income	**0.917**	0.906–0.930	**0.939**	0.929–0.950
Physical activity	0.994	0.982–1.006	0.997	0.987–1.007
Sport volume (hours)	0.999	0.998–1.000	**0.998**	0.997–0.999
Team sports	1.008	0.921–1.103	1.051	0.974–1.134
Individual sports	1.119	0.996–1.258	1.070	0.968–1.183
Team + individual sports	1.105	0.980–1.246	1.104	0.998–1.222
Sex * team sports	**0.797**	0.683–0.929	0.951	0.847–1.069
Sex * individual sports	0.896	0.769–1.045	0.974	0.858–1.104
Sex * team + individual sports	**0.832**	0.702–0.986	0.932	0.815–1.066
**Variance**				
Conditional R^2^	0.089		0/042	

*Note*. Statistically significant values (*p* < .05) are in bold.

## Discussion

The purpose of this study was to explore the association between participation in organized sport and a broad collection of mental health difficulties with a large sample of US children and adolescents. Main findings were that children and adolescents who participated in team sport had fewer mental health difficulties across a range of syndromes compared to those who did not participate in organized sport. Children and adolescents who participated in individual sport had greater levels of mental health difficulties across several syndromes compared to those who did not participate in organized sport. In general, children and adolescents who participated in both team and individual sport did not demonstrate significant differences in terms of mental health difficulties compared to non-sport participants. The results suggest that type of sport may be a salient factor in the relationship between sport participation and mental health.

Consistent with our hypothesis, children and adolescents who were involved in organized team sport experienced a lower prevalence of mental health difficulties compared to those not engaged in organized sport. Specifically, children and adolescents who played team sports were less likely to experience symptoms of anxiety and depression, withdrawal, social problems, thought problems, attentional problems, and rule-breaking behavior (among females only). These findings align with previous work highlighting the benefits of team sport participation for youth’s mental health,(e.g., [13,15]) but also add to emerging knowledge concerning the emotional and behavioral benefits that coincide with playing organized sport in a team setting. Using the Strengths and Difficulties Questionnaire (SDQ) [33], which is conceptually similar to the CBCL, recent research showed that greater team sport participation related to fewer emotional symptoms, conduct problems, peer problems, and hyperactivity among Australian adolescents (12–17 years) [13]. Further, greater team sport participation inversely predicted emotional symptoms at later timepoints in adolescence (and vice versa), but did not prospectively predict the remaining mental health indices from the SDQ. Regardless, as previously noted, the body of literature in this area seems to indicate at least a consistent correlational relationship between team sport participation and fewer mental health difficulties. Team sport participation is thought to be particularly effective in protecting against the development of mental health problems among children and adolescents because of the many opportunities for positive social interactions [3]. It may also be that children and adolescents who compete in organized team sport experience a sense of closeness and cohesion with their teammates (i.e., feeling connected with team members on a task and social level) [34], which may be beneficial for their mental health. Indeed, perceptions of team cohesion have been linked to the development of social skills [35] and stronger beliefs in one’s ability to cope with stress [36].

In contrast to our hypothesis, children and adolescents who played strictly individual sports experienced a greater prevalence of mental health difficulties compared to their non-sport playing counterparts. Individual sport participants demonstrated greater anxiety and depression, withdrawal, social problems, and attentional problems. Although research findings suggest individual activities may not offer mental and psychosocial health benefits to the same extent as team sports [3,11,14,22,23], to our knowledge little to no research has reported that sport played independently may in fact be problematic for mental health relative to non-sport participation. It is possible that some children and adolescents who compete in individual sports experience significant stress associated with performing independently, which could contribute to mental health problems [37]. The potential for enhanced self-awareness and pressure that stems from performing a task independently in front of a supportive audience has been well-documented [38]. It is conceivable that children and adolescents competing individually would be keenly aware of the performance expectations (real or perceived) placed on them by their parents/guardians, family, or peers, which could result in pressure to perform well. Individual sport athletes are also prone to attribute their failures to internal factors, presumably because they do not have teammates with whom they can share the blame for perceived poor performances [39,40]. One can certainly appreciate how assuming all responsibility for sport-related failures could contribute to decreased mental well-being. Additionally, individual sport athletes in particular may be susceptible to social physique anxiety (i.e., fear of negative evaluation by others regarding one’s body appearance) [41]. Pressure from the threat of negative body appraisals by others would be particularly relevant to children and adolescents in our study who competed in body-focused sports (e.g., ballet/dance), which might partly explain why individual sport athletes were more likely to experience symptoms of anxiety and depression compared to non-sport participants.

Contrary to our findings, other studies have revealed positive associations between individual sport participation and mental health outcomes. Compared to non-sport participation, a greater number of years competing in individual sport (or team sport) in adolescence related to fewer panic symptoms in young adulthood; a greater number of years playing only individual sports in adolescence related to fewer social phobia symptoms in young adulthood [17]. In another study, children who participated in individual sport (or team sport) exhibited fewer inattention/hyperactivity problems, and children who competed in only individual sport had lower ‘acting without thinking’ scores, compared to children who did not participate in sport [16]. Thus, our finding that individual sport participants may be at increased risk for mental health difficulties (relative to non-sport participants) requires more investigation. Future ABCD data releases will allow for a more thorough understanding of child and adolescent participation in individual sport and its relationship to mental health difficulties later in life.

It can perhaps be assumed that the benefits of team sport and detriments of individual sport on mental health difficulties cancelled each other out in our analysis of both forms of sport participation relative to non-sport participation. The only significant finding for this sport category was that participation in both team and individual sport was associated with lower rule-breaking behavior for females (compared to males). Moreover, beyond rule-breaking behavior, sex was not a significant moderating factor across the remaining models. Although there was scant evidence of sex effects in this study, recent meta-analytic results demonstrated a stronger negative relationship between sport participation and anxiety in studies with comparatively more male than female participants (mean age: 12–18 years) [4]. Another recent study with adolescent athletes (12–17 years) revealed that males who participated in more team sport tended to report fewer depressive symptoms (but did not report fewer depressive symptoms at later timepoints), whereas females who were involved in more team sport did not tend to report fewer depressive symptoms (but did report fewer depressive symptoms at later timepoints) [13]. In sum, additional research exploring potential sex differences is encouraged, particularly with respect to emotional and behavior difficulties among children and adolescents.

The results of this study, coupled with previous research findings, suggest that participation in organized team sport may be a useful medium through which to promote child and adolescent mental health. Efforts to provide children and adolescents with affordable options to join organized team sport leagues/clubs outside of school may require further attention, particularly for families with socioeconomic challenges [7]. Opportunities to improve the mental health of children and adolescents already enrolled in organized sport should also be explored. One such approach might involve exposing young sport participants to mental health literacy (i.e., awareness) programs within the sport context, where they might feel comfortable enhancing their understanding of mental health among relatable peers. Results of a sports-based mental health literacy intervention with male adolescents aged 12–18 years showed that those in the intervention group demonstrated improvements in mental health literacy for signs and symptoms of depression and anxiety, intentions to help others potentially suffering from mental health problems, and help-seeking attitudes [42]. Adolescents who participated in the mental health literacy intervention also described in focus groups that the program enhanced their mental health knowledge, as well as their confidence and intentions to both provide and seek help [43]. These studies offer promising results for the effectiveness of sports-based mental health literacy programs, particularly for young males who have more negative views than females regarding mental health treatment [44] and whose sport coaches may encourage masculine norms [45] that potentially undermine favorable help-seeking attitudes. Nonetheless, evidence-based mental health awareness programs for sport-specific populations remain limited [46], and thus further work in this area is needed. Given the results of the present study, implementing mental health literacy programming with individual children and adolescent sport athletes may be particularly warranted.

When interpreting the results of this study, readers might consider some limitations. Given the cross-sectional design, we cannot make causal claims regarding the relationship between participation in organized sport and mental health difficulties. That is, the results do not shed light on whether sport participation facilitates child and adolescent mental health or whether mental health scores predict whether children and adolescents are more or less likely to participate in different types of sports. The bidirectional nature of the relationship between sport participation and youth mental health has been demonstrated [31], and researchers have explored the association between sport participation and mental health from adolescence to young adulthood [15,17,23]. Nonetheless, intensive longitudinal work (i.e., more than two time points) that can address causality and causal mechanisms in the sport-mental health relationship is needed [5], and can be explored with future ABCD releases. Another potential limitation is that child and adolescent mental health difficulty scores in this study were based on parent-reports. It is possible that parents/guardians who enroll their children in individual sports tend to overestimate the problematic aspects of their children’s emotional and behavioral conduct. Alternatively, it is plausible that parents/guardians who register their children in team sports are more likely to view their children interacting positively with peers and thus tend to underestimate any potential mental health difficulties. On a related note, as there were generally fewer mental health difficulties among females compared to males, it is possible that some parents may be insensitive to the kinds of mental health problems experienced by young females. It would be useful to confirm the results of this study using child and adolescent self-report data. Lastly, the obtained effect sizes in this study were relatively small (10–20% increases/decreases in mental health difficulty scores).

Despite these limitations, this study has several strengths. Namely, the large, broadly representative sample instills confidence that the results are relevant to children and adolescents across the United States. These results provide researchers, practitioners, and public health officials with up-to-date information concerning the relationship between organized youth sport participation and mental health difficulties. Further, the mental health outcomes in this study move beyond traditional indices (e.g., depression) and add to emerging research focused on emotional and behavioral difficulties among youth [12,13,31]. Additionally, our analyses included several covariates, including overall physical activity levels and sport volume, which have not been consistently controlled for in previous research. Physical activity scores were, however, collected at baseline and thus served only as an estimate of participants’ physical activity levels at wave 2.

To advance the literature in this area, future research might consider assessing mental health outcomes in relation to more distinct team sport types. For example, researchers have noted that team sports can be classified into one of six sport types (e.g., segregated, cooperative) based on their level of task interdependence and emphasis on group versus individual outcomes [47]. Assessing distinct team sport types would require sufficient sample sizes across several categories of team sports but would provide a more nuanced understanding of the relationship between youth sport participation and mental health. Future research might also consider exploring (and controlling for) parent/guardian as well as youth’s knowledge and understanding of mental health. As the topic of mental health continues to garner attention and becomes more mainstream, research participants may be more acutely aware of mental health concerns, have the language to discuss them, and feel increasingly comfortable reporting their own mental health difficulties or sharing their views on the mental health of others. Finally, future studies could examine the link between sport participation and mental health among minority youth populations (e.g., LGBTQ, BIPOC) who may be at increased risk for mental health difficulties.

## Conclusion

As reflected by parent-reports, participation in exclusively team sport (compared to no sport participation) was associated with fewer mental health difficulties, whereas participation in exclusively individual sport (compared to no sport participation) was associated with greater mental health difficulties. Generally, athletes who played both team and individual sport did not have different mental health profiles compared to non-sport playing children and adolescents. The findings complement previous work suggesting that team-based organized sport may be a vehicle to support youth mental health. Future research should confirm to what extent, and under what circumstances, participating in strictly individual sport may be problematic for child and adolescent mental health outcomes.

## Supporting information

S1 Fig Spearman correlations among major study variables. Values are significant at *p* < .05. Missing values are non-significant.
(DOCX)
