# Supplementary material for: Associations between organized sport participation and mental health difficulties: Data from over 11,000 US children and adolescents
Source: PLoS One. 2022 Jun 1;17(6):e0268583. doi: 10.1371/journal.pone.0268583 (PMC9159603; doi:10.1371/journal.pone.0268583)
Supplement: S1 Fig — Values are significant at p < .05. Missing values are non-significant. (DOCX) [file pone.0268583.s001.docx]

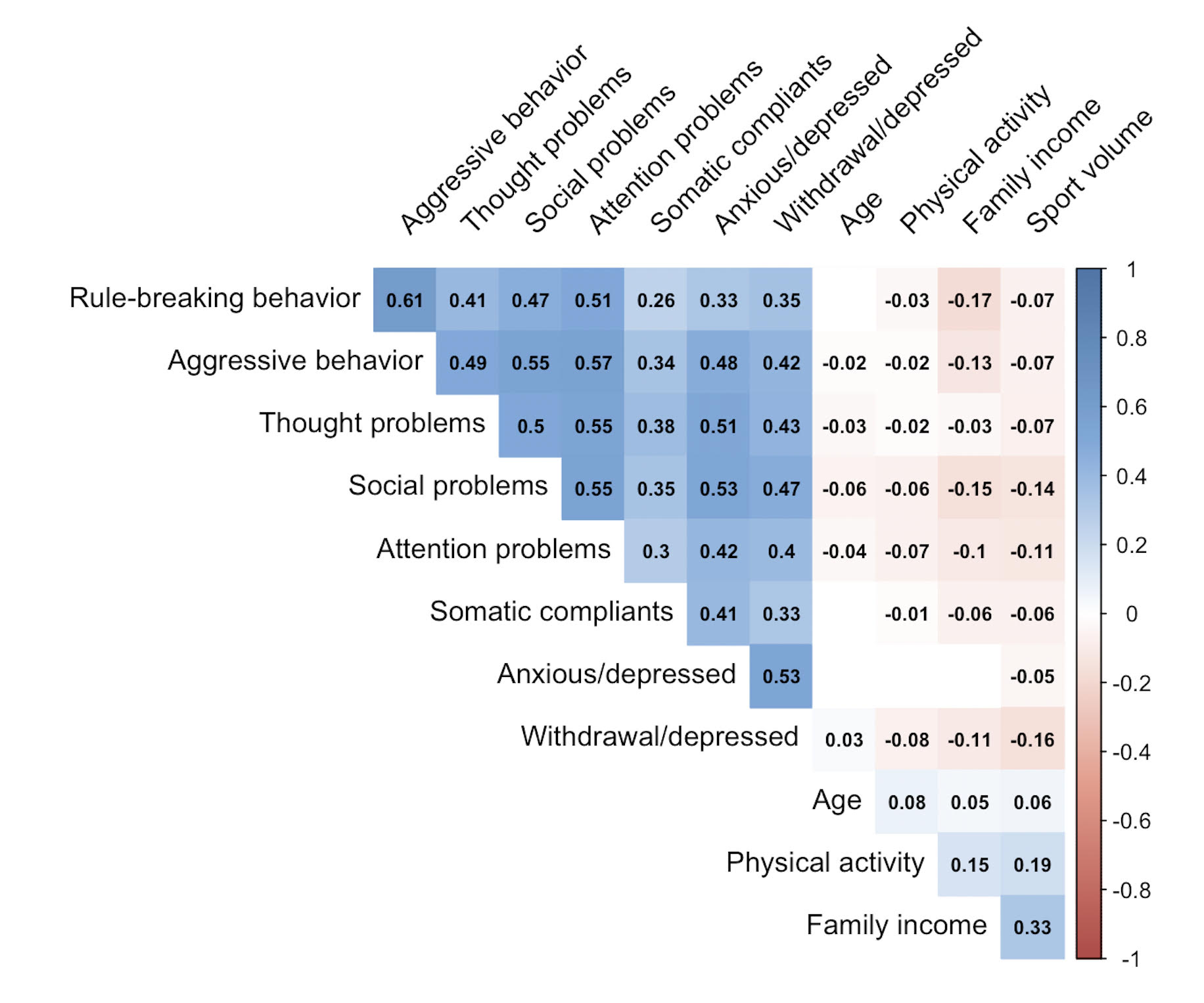


**S1 Fig.** **Spearman correlations among major study variables.** Values are significant at *p*<.05. Missing values are non-significant.
